# Assessment of physical activity, capacity and nutritional status in elderly peritoneal dialysis patients

**DOI:** 10.1186/s12882-017-0593-7

**Published:** 2017-05-30

**Authors:** Adamasco Cupisti, Claudia D’Alessandro, Viviana Finato, Claudia Del Corso, Battista Catania, Gian Marco Caselli, Maria Francesca Egidi

**Affiliations:** 1 Department of Clinical and Experimental Medicine, University of Pisa, Via Roma 67, 56126 Pisa, Italy; 2 Nephrology and Dialysis Unit, S. Miniato Hospital, S. Miniato, Italy; 3 Nephrology and Dialysis Unit, Pistoia Hospital, Pistoia, Italy; 4 Nephrology and Dialysis Unit, Pontedera Hospital, Pontedera, Italy; 5 Nephrology and Dialysis Unit, S. Giovanni di Dio Hospital, Florence, Italy

**Keywords:** Physical activity, Nutritional status, Peritoneal dialysis, Esrd, MIS, Rapa, CKD, Performance

## Abstract

**Background:**

This study aimed to evaluate the prevalence of sedentarism, and to assess physical capacity and nutritional status in a cohort of older patients on peritoneal dialysis *(*PD), with respect to age-matched non-dialysis CKD population, using highly accessible, simple methods, namely the Rapid Assessment of Physical activity (RAPA) test and the 30″ Sit-to-stand (STS) test.

**Methods:**

This cross-sectional multicenter study included 151 renal patients older than 60 years; 71 pts. (44 m, age 72 ± 7 yrs) were on PD and 80 pts. (63 m, age 74 ± 7 yrs) were affected by 3–4 stage CKD.

**Results:**

The prevalence of sedentary/underactive patients was double of that of the active patients as assessed by RAPA test, both in the PD (65.3%) and in the CKD (67.5%) cohort.

The 30"STS test showed a reduced physical performance in both groups: 84.5% of PD patients and 87.5% of CKD patients did not reach the expected number of stands by age and gender. A malnutrition-inflammation score (MIS) ≥ 6 occurred in 37 % of PD patients and in 2.5 % of CKD patients. In PD patients, an independent significant association was observed between 30”STS test and MIS (beta -0.510, *p* = 0.013), as well as between RAPA and MIS (beta -0.544, *p* = 003) and phase angle (beta -0.506, *p* = 0.028).

**Conclusions:**

A high prevalence of low- performance capacity and sedentarism has been detected among elderly patients on PD or with CKD stage 3–4. Apart from age, a condition of malnutrition-inflammation was the major determinant of poor physical activity and capacity in PD patients. Better body composition seems to be positively associated with physical activity in PD and with physical capacity in CKD patients. Routine clinical management should include a close evaluation of nutritional status and evaluation of physical activity and capacity which can be easily assessed by RAPA and 30″STS tests.

## Background

The treatment of chronic kidney disease (CKD) aims to reduce progression of renal and cardiovascular damage, so as to prevent uremic complications and to improve survival. However, new challenges must be addressed to ameliorate the health status and to prevent disability. A proper nutritional approach and regular physical activity are important to improve the quality of life and to maintain physical performance and ability in renal patients [1].

Physical inactivity is a long-standing clinical problem among pre-dialysis CKD patients [2] and end-stage renal disease (ESRD) patients on dialysis treatment [3]. It contributes to disability and to poor nutritional status that is associated with increased morbidity and mortality risk.

Evidence exists of an association between sedentary behavior and mortality in ESRD patients [4, 5]. In survival analysis, sedentary behavior was associated with an increased risk for death at 1-year follow-up after adjusting for all the variables linked to the mortality risk [6].

Advancing age is another major determinant of physical functioning and a sedentary lifestyle is largely prevalent in elderly people, including renal patients. Protein energy wasting (PEW) is another condition that is associated with disability, frailty and low physical activity and capacity [7]. PEW is quite prevalent in CKD and ESRD patients, including PD patients [8]. Since the PD is a suitable method for the increased amount of elderly patients with ESRD [9], nutritional and physical functioning evaluation is an outstanding aspect of the clinical management.

The aim of the study was to evaluate the prevalence of low physical activity, and to assess physical capacity and nutritional status in a cohort of PD older patients with respect to age-matched non-dialysis CKD population, using highly accessible, simple methods. The determinants of hypo-activity were also investigated.

## Methods

This is a cross-sectional case-control multicenter study that included 151 prevalent renal patients aged older than 60 years; 71 pts. (44 m, 27 f, age 72 ± 7 yrs) were on PD by 33 ± 20 months, and 80 pts. (63 m, 17 f, age 74 ± 7 yrs) affected by CKD stages 3–4 were on tertiary care management. Five peritoneal dialysis units sited in Tuscany (Pisa, Pontedera, S. Miniato, Pistoia and Florence) participated in the study.

Patients with physical or neurological disability, muscular diseases or lower-limb amputation were excluded as well as those with acute illness or infections, severe heart or respiratory insufficiency, peritonitis or patients on CKD stage 5-ND.

All the patients underwent an evaluation of their physical function and nutritional status in an out-patients clinic setting, during the period from 1st January to 31st December 2015.

Spontaneous physical activity and physical performance were detected using the rapid assessment of physical activity (RAPA) and the 30” Sit-to-Stand (30” STS) chair test, respectively.

The RAPA is a validated test able to assess habitual physical activity in adults older than 50 years [10]. The RAPA was administered to the patients before the visit to the renal out-patient clinic. RAPA test score ≤ 3 corresponded to a sedentary lifestyle or a very light activity level; values ≥4 indicated a moderate to vigorous active lifestyle [10].

The 30” STS chair test is a validated test able to assess lower extremity strength in adults older than 60 years [11]. In the 30” STS chair test the participant is seated in the chair with his/her arms crossed and held against the shoulders. The score correspond to the number of stands a person can complete in 30” without the help of arms. Data were compared also with standard values for age and gender [11, 12].

Anthropometry was performed at the out-patient clinic, with empty abdomen in PD patients. It included measurements of body weight, height, triceps skinfold thickness and body circumferences such as middle arm, hip and waist [13]. Body mass index, and middle arm muscle circumference were calculated. As body composition analysis parameters, we reported phase angle and Body mass cell index [14] detected by Bio-Impedance Analysis (BIA). Phase angle <5.0° in men and <4.9° in women have been assumed as cut-off values for risk of under-nutrition [15].

Biochemical evaluation was performed after an overnight fasting and included: urea, creatinine, electrolytes, bicarbonate, albumin, 25(OH)VitD serum levels and hemoglobin. Renal function in CKD patients was reported as estimated GFR (eGFR), using CKD-EPI creatinine formula.

Demographic and clinical data, and comorbidities were extracted by patient’s medical chart. Charlson comorbidity index (CCI) was calculated accordingly.

Malnutrition Inflammation Score [16, 17], was calculated as follows: scores 6–10 were indicative of mild malnutrition and score ≥ 11 were indicative of severe malnutrition. An appetite questionnaire, namely the council of nutrition appetite questionnaire (CNAQ), was self-administered [18]. The Geriatric Nutritional Risk Index (GNRI) is a very simple and objective method to assess nutritional status in a number of pathological conditions, including dialysis patients [19–21]. It is based on body weight, height and serum albumin levels. The GNRI was calculated by modifying the Nutritional risk index for elderly patients [22] as follows: GNRI = [14.89 * albumin (g/dl)] + [41.7 * (body weight/ideal body weight)]. As body weight we considered the value in the morning, at empty abdomen: it was also the value used for body mass index (BMI) calculation (expressed as kg/m^2^); body weight/ideal body weight was set to 1 when the patient’s body weight exceeded the ideal body weight [5]. The ideal body weight in the present study was assumed as the value calculated from the height to obtain a BMI of 22.5 kg/m^2^, which is the reference for the Italian population.

Patients gave their informed consent to participate in the study which was in accordance with the Helsinki declaration. The study protocol was approved by the Local Ethics Committee of Pisa University Hospital.

### Statistical analysis

Data are expressed as mean ± standard deviation (SD) or median and inter-quartile range (IQR) when appropriate. Comparisons between groups were assessed by Mann-Whitney U test for independent samples. Chi-square test was used for analysis of frequencies. Spearman linear correlation analysis was used to determine associations between various selected parameters.

Multivariate statistical analysis was conducted for examining the effect of multiple independent variables, namely age, gender, Charlson Comorbidity Index, Appetite Score. Malnutrition Inflammation Score, phase angle and BCMI on physical capacity assessed by the 30” STS chair test or on physical activity assessed by RAPA test.

Statistical evaluation was performed using IBM SPSS statistics version 19 (SPSS Inc., Illinois, USA) for Windows.

Differences were considered as statistical significant when *p* < 0.05.

## Results

Clinical features of PD and CKD patients are reported in Table 1.

**Table 1:** Clinical features of peritoneal dialysis (PD) and chronic renal disease (CKD) patients. Data are reported as Median (IQR)

	PD	CKD
	*n* = 71	*n* = 80
Age, years	73.0 (68.0–76.0)	75.0 (69.0–80.8)
Body weight, Kg	72.5 (62.5–87.0)*	78.0 (71.6–87.3)
BMI, Kg/m^2^	26.0 (22.4–29.4)*	27.6 (25.3–30.9)
Charlson Index	7.0 (5.0–8.0)	7.0 (6.0–8.0)
MIS	5.0 (3.0–6.0)***	0 (0–2.0)
Appetite (CNAQ)	29.0 (25.0–32.0)**	31.0 (29.0–32.0)
GNRI	95.7 (90.8–100)***	103 (98.5–107)
RAPA	3.0 (2.0–4.0)*	2.0 (0–5.0)
30″ STS, n.stands	10.5 (8.8–13.0)	10.0 (8.0–12.0)
sUrea, mg/dl	124 (105–150)***	81.1 (61.0–97.0)
sCreatinine, mg/dl	7.8 (5.8–9.2)***	2.0 (1.5–2.8)
sAlbumin, g/dl	3.6 (3.3–3.8)***	4.2 (3.9–4.4)
Haemoglobin, g/dl	11.5 (10.8–12.2)***	13.0 (11.8–14.7)
sPhosphate, mg/dl	4.7 (3.9–5.3)***	3.3 (2.9–3.7)
sCalcium, mg/dl	9.2 (8.7–9.4)*	9.3 (9.1–9.6)
sSodium, mEq/l	140 (137–142)**	141 (139–142)
sPotassium, mEq/l	4.2 (3.8–4.9)***	4.6 (4.4–5.1)
sBicarbonate, mM	25.2 (23.1–28.0)*	24.4 (22.0–26.7)
25OH Vit D, ng/ml	13.4 (8.6–23.2)	21.9 (15.4–28.1)

*GNRI* geriatric nutrition risk index, *MIS* malnutrition inflammation score, *CNAQ* council of nutrition appetite questionnaire, *BMI* body mass index, *GNRI* geriatric nutrition risk index; 30″STS: 30 “Sit-to-Stand

* *p* < 0.05, **P < 0.01, ***P < 0.001 vs CKD

Thirty-six PD patients were on continuous ambulatory peritoneal dialysis (CAPD) (50.7%) and 35 (49.3%) were on automated peritoneal dialysis (APD). Weekly Kt/V in the PD group resulted 2.0 ± 0.5. In the CKD group, eGFR was 32 ± 12 ml/min*1.73 m^2^, range 16–59 ml/min*1.73 m^2^.

As expected, PD patients had more pronounced metabolic abnormalities and serum albumin markedly lower than CKD patients. Hemoglobin level was lower in PD patients but it was well in accordance with the current recommendations. A good control of serum potassium and of metabolic acidosis was detected in PD group, while a vitamin D deficiency was quite prevalent in both groups [Table 1].

**Fig. 1:**
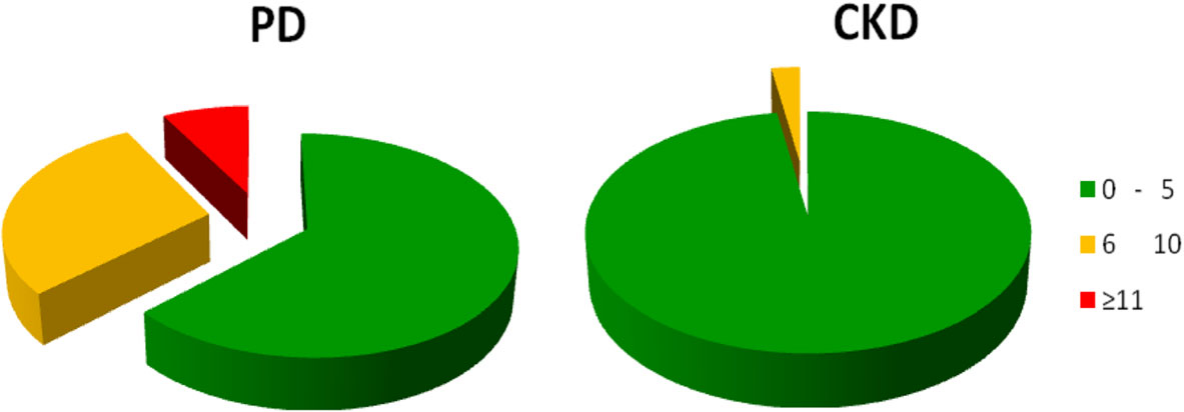
Malnutrition Inflammation Score in the Peritoneal Dialysis (PD) and in the chronic kidney disease (CKD) patients cohorts. A score 0–5 is indicative of normal nutrition (*green*); 6–10 score is indicative of mild malnutrition (*orange*), while score ≥ 11 is suggestive of moderate to severe malnutrition (*red*)

The CCI indicated a very similar level of comorbidity. Instead, MIS was markedly higher in PD than in CKD patients (Table 1). A MIS score suggestive of malnutrition (≥6) occurred in 37% of PD patients and 2.5% of CKD patients (Fig. 1). A GNRI score < 92, as suggestive of a poor outcome, was found in 31% of PD patients and 3.8% of CKD patients (*p* < 0.001). In Table 2 some nutritional aspects of PD and CKD patients are reported, by gender.

**Table 2:** RAPA and 30″STS test, and parameters of nutritional status in peritoneal dialysis (PD) and chronic kidney disease (CKD) patients by gender. Data are reported as Median (IQR)

	Males		Females	
	PD *n* = 44	CKD *n* = 63	PD *n* = 27	CKD *n* = 17
Age, years	73.0 (66.3–76.0)	75.0 (69.0–81.0)	70.0 (64.0–76.0)	75.0 (66.0–78.5)
Body weight, Kg	78.6 (71.6–90.4)	78.6 (72.7–88.8)	61.3 (55.7–72.0) *	70.0 (64.3–83.3)
BMI, Kg/m^2^	26.7 (22.7–29.9)	27.5 (25.5–30.7)	24.0 (22.0–28.2)	28.2 (24.8–33.7)
RAPA Test 1	3.0 (2.0–4.8)	2.0 (0–5.0)	2.0 (1.0–4.0)	2.0 (0.5–2.0)
Sit-to-stand (30 “), n. stands	10.0 (9.0–12.0)	11.0 (8.0–13.0)	11.0 (8.0–13.0)	8.0 (7.0–10.0)
Waist Circumference, cm	105 (98.0–119)	103 (96.5–109)	96.0 (85.0–102)	97.0 (88.0–107)
Hip Circumference, cm	107 (100–115)	104 (99.0–109)	98.0 (94.0–106) *	109 (102–114)
TST, cm	1.0 (0.6–2.1)	1.0 (0.7–1.3)	0.7 (0.4–1.0) *	1.8 (1.1–2.3)
MAC, cm	29.0 (27.0–31.0)	29.0 (27.0–32.0)	27.0 (25.2–31.0)	29.7 (26.1–34.0)
MAMC, cm	26.3 (22.4–28.0)	25.8 (24.3–27.9)	25.6 (22.2–29.5)	25.0 (21.2–28.6)
Phase angle, °	5.5 (4.3–6.2)	4.9 (4.3–5.5)	5.0 (4.2–5.4)	4.3 (4.1–4.9)
BCMI, Kg/m^2^	8.5 (7.6–10.3)	9.2 (8.1–10.5)	7.3 (6.1–8.4)	7.7 (7.0–8.2)

*TST* triceps skinfold thickness, *MAC* middle arm circumference, *MAMC* middle arm muscle circumference, *BMI* body mass index, *RAPA* rapid assessment of physical activity, *TST* triceps skinfold thickness, *BCMI* body cell mass index

* *p* < 0.05 vs CKD

The reported level of RAPA score was similar in PD e CKD patients (Tables 1– 2).

**Fig. 2:**
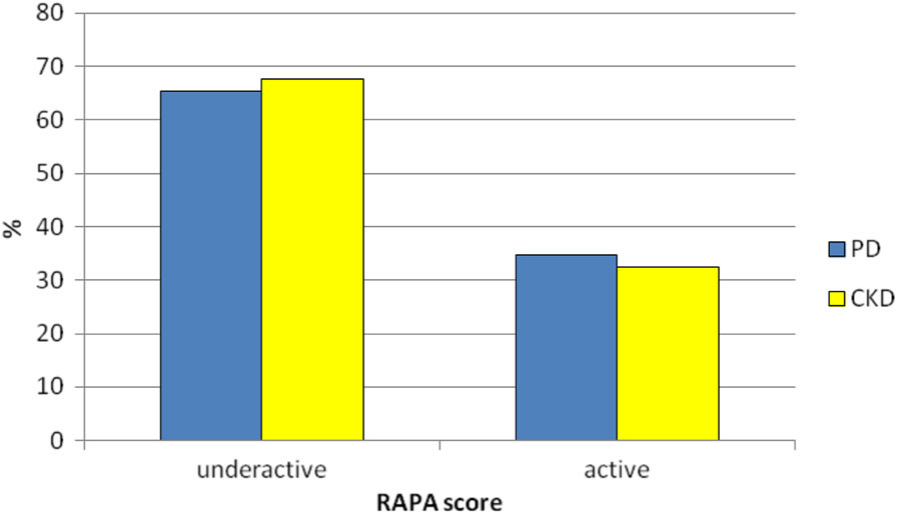
Prevalence of sedentary/underactive or moderate to vigorous active patients as assessed by RAPA test (score ≤ 3 or ≥4, respectively) in the PD and CKD studied cohorts

The prevalence of sedentary/underactive patients was roughly double than that of active patients as assessed by RAPA. Namely, 65.3% of PD patients and 67.5% of CKD patients resulted sedentary/underactive by the RAPA test; no difference was detected between PD and CKD groups (Fig. 2).

When compared to PD patients who reported moderate to vigorous physical activity, sedentary/underactive PD patients were older and had a higher MIS score, lower appetite score, lower GNRI, worse performance at the STS test, and a reduced middle arm muscle circumference (Table 3). Phase angle values <5.0° in men or <4.9° in women were detected in 50% of patients of the under-active group and in 18% of the active group.

**Table 3:** Differences between PD patients assessed as sedentary/underactive or moderately to vigorously physically active by RAPA test scoring. Data are reported as Median (IQR)

	Underactive	Active	*p*
Age, years	75.0 (69.8–79.0)	68.0 (63.0–73.0)	<0.001
BMI, Kg/m^2^	25.8 (22.0–29.0)	26.0 (22.9–30.5)	n.s.
MIS	6.0 (4.0–7.5)	3.0 (2.0–4.0)	<0.001
GNRI	95.3 (90.5–98.1)	98.7(95.3–102)	<0.05
Appetite, CNQA	28.0 (23.8–31.0)	30.0 (28.5–32.0)	<0.01
sUrea, mg/dl	120 (109–144)	136 (98.7–159)	n.s.
sCreatinine, mg/dl	7.7 (5.8–9.2)	8.0 (5.9–9.7)	n.s.
sAlbumin, g/dl	3.6 (3.3–3.7)	3.7 (3.3–3.9)	n.s.
Haemoglobin, g/dl	11.5 (10.8–12.1)	12.0 (10.0–14.0)	n.s.
30″STS, n. stands	10.0 (6.5–12.0)	12.0 (10.0–14.0)	<0.01
Waist circumference, cm	101 (92.5–111)	100 (87.0–115)	n.s.
MAC, cm	28.5 (25.9–31.0)	30.0 (26.5–33.5)	n.s.
MAMC, cm	24.0 (22.0–27.6)	27.5 (25.6–29.0)	<0.05
Phase angle, °	4.9 (3.7–5.9)	5.4 (5.0–6.2)	n.s.
BCMI, Kg/m^2^	7.9 (6.7–9.5)	8.2 (7.3–10.5)	n.s

*PA* physical activity, *BMI* body mass index, *MAC* middle arm circumference, *MAMC* middle arm muscular circumference, *GNRI* geriatric nutrition risk index, *MIS* malnutrition inflammation score, *CNAQ* council of nutrition appetite questionnaire, *BCMI* body cell mass index

**Fig. 3:**
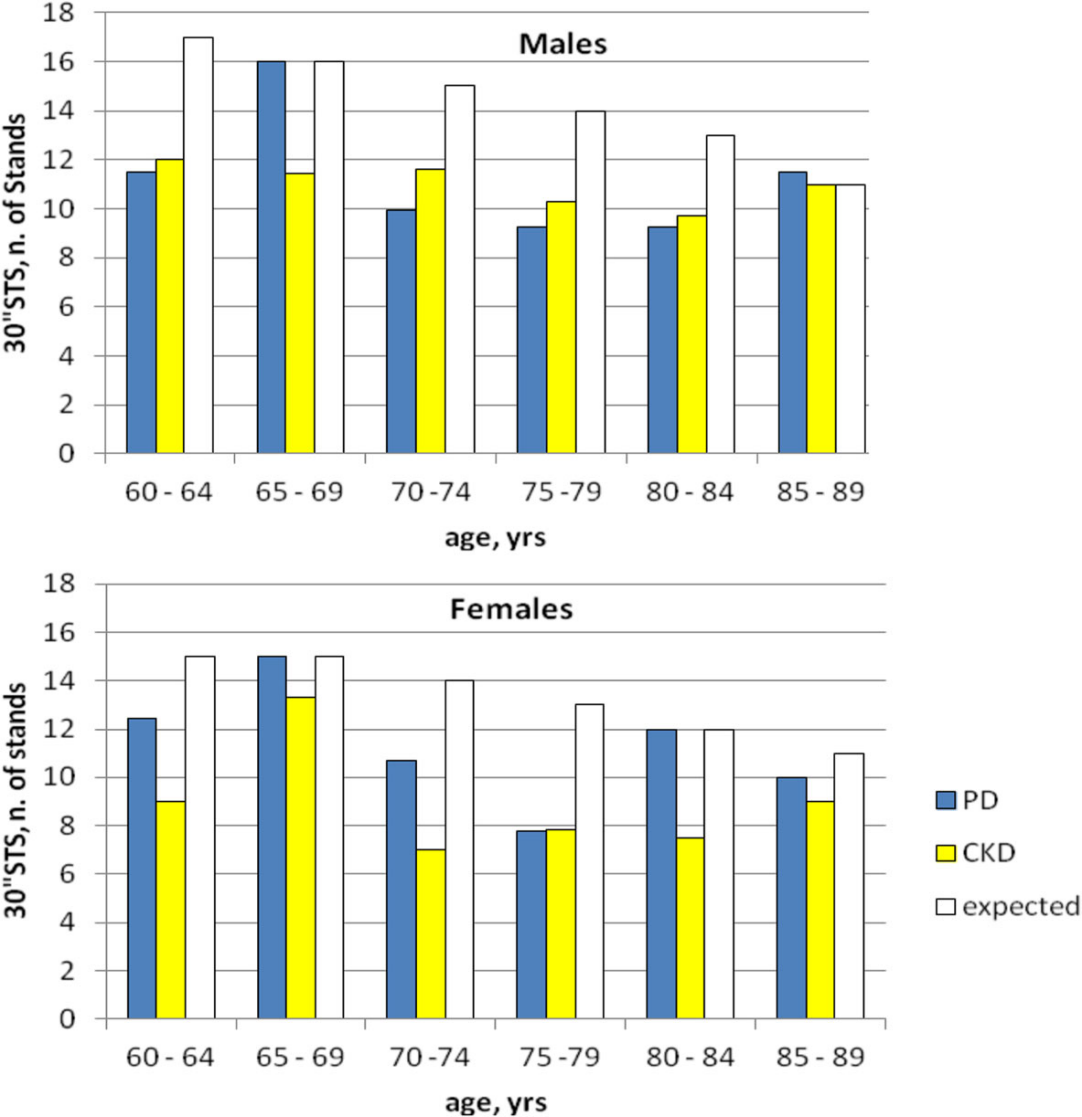
Average number of stands to the 30″ sit-to-stand (30”STS) test in males and females. The empty column represents the number of stands (by sex and age) predictive of physical activity maintenance

The results of the 30″STS tests were not different between CKD and PD patients (Tables 1– 2). As a whole, the age-related average number of repetitions to the 30” STS test resulted lower than expected both in males and females, but similar between PD and CKD patients (Fig. 3). 84.5% of PD patients and 87.5% of CKD patients did not reach the expected number of stands by age and gender to maintain physical activity.

No difference was detected between patients on CAPD and APD as far as nutritional status, RAPA or 30″STS test were concerned.

In PD patients linear correlation analysis showed that the STS performance negatively correlated to age (*r* = −0.295, *p* < 0.05) and MIS (*r* = −0.424, *p* < 0.01) and positively to appetite score (*r* = 0.255, *p* < 0.05). The same significant correlations were observed for the RAPA score (age: *r* = −0.413, *p* < 0.01; MIS: *r* = −0.595, *p* < 0.01; appetite score: *r* = 0.395, *p* < 0.01).

In CKD patients STS performance was negatively associated with MIS (*r* = −0.255, *p* < 0.05).

Multivariate statistical analysis was performed to analyze the effect of multiple independent variables, namely age, gender, Charlson Comorbidity Index, Appetite Score, MIS, phase angle and BCMI, on physical performance assessed by the 30″ STS chair test.

In PD patients, an independent significant association was observed between 30”STS test and MIS (beta −0.510, *p* = 0.013), as well as between RAPA and MIS (beta −0.544, *p* = 0.003) and phase angle (beta −0.506, *p* = 0.028).

In CKD patients the multivariate analysis showed an independent significant association between 30”STS test and gender (beta −0.276, *p* = 0.020) and BCMI (beta −0.328, *p* = 0.009).

## Discussion

The results of our study indicate that PD patients are largely inactive as detected by RAPA test and have a low-level of physical performance as detected by the 30”STS test. This occurred when data were compared to regular standards. However, when performance level of PD patients were compared with the cohort of non-dialysis CKD patients no difference emerged.

It suggests that peritoneal dialysis per se’ is not a major cause of worsening of physical activity and performance. Instead, malnutrition-inflammation was the major determinant of physical impairment in our PD cohort, where higher physical activity was associated with better body composition.

A number of studies addressed physical functioning in ESRD patients but few papers have been focused on physical activity and performance in PD patients, especially in the elderly cohort.

Our data are well in keeping with those of Cobo et al., who reported a high prevalence of sedentary lifestyle (63%) in a cohort of 64 PD patients as assessed by pedometers [23]. They found that better physical activity was associated with better nutritional status and lower comorbidity [23]. It is noteworthy that those patients were approximately 10 years younger than our cohort.

Pedometers and other wearable devices such as the sense-wear arm-band [24, 25] are useful and reliable tools to assess physical activity level in the daily life. However, limited resources or poor patient’s compliance may prevent a widespread applications of these devices in the clinical management of CKD and ESRD populations. RAPA and 30”STS have the advantages to be simple, less time consuming, inexpensive and feasible in all clinical settings.

The RAPA test has been validated as a tool for the assessment of physical activity in adults older than 50 years [10]. It is very simple test, self-administered, and not time-consuming. It has all the limits of the self-reporting test, but it may be very useful for an extensive routine implementation in the daily life clinical practice. It has been favorably tested also as a telephone-based questionnaire [26].

Similarly, 30″ Sit-to-Stand test [11] is a validated test able to assess functional lower extremity strength in adults older than 60 years. It is easy to perform everywhere and takes very little time. The scoring is predictive of functional capacity and is related to nutritional status and prognosis in several chronic diseases [12].

Malnutrition-inflammation seems to be one of the major determinant of physical impairment in our PD cohort. Moreover, this condition may be favored by the high protein loss, and by the abdominal discomfort and glucose load that may induce loss of appetite, that is quite prevalent in PD patients.

The negative relationship between STS test and MIS and between RAPA test score and MIS observed in our PD patients is of interest and it is in accordance with other reports. MIS was found to be a reliable prognostic predictor [16], which was associated to the level of physical performance and functional capacity of PD patients [27].

It has been reported that dialysis patients with laboratory-based evidence of malnutrition and/or inflammation were likely to report lower levels of physical activity [28, 29]. It is noteworthy that exercise could evoke anti inflammatory effects in predialysis CKD patients [30].

In the PD patients, physical activity was associated with better body composition. Similarly, in the CKD group, physical performance was positively related to higher body cell mass index. Although these results may be quite expected and do not demonstrate causality, they confirm that body composition and functional aspects are closely related and inter-dependent in renal patients.

Moreover, favorable effects of physical activity implementation have been described [3] including psychological and health perception effects [31]. This may be a further reason to stimulate physical exercise programs in the ESRD population [32].

The relationship between nutritional status and physical activity is of special value in PD patients who are, on one side, at high risk of protein depletion, and on the other side at risk of obesity [8, 33]. In this clinical setting, implementation of a regular physical activity could contribute to maintain muscle mass and to increase energy expenditure.

Unfortunately, many barriers prevent a regular assessment of physical activity and implementation of exercise programs in ESRD patients [34, 35].

Studies have shown a progressive decline of physical function with declining GFR, and cross-sectional and observational studies have shown relationships between declining GFR and physical function, muscle mass and performance [36–38]. In the present study, we failed to find a relationship between eGFR and physical activity level, and no major differences in physical activity and capacity were detected between CKD and PD patients. This discrepancy may be justified, in part at least, by the fact that our patients were already on tertiary care and received standard nutritional and pharmacological therapies, consequently most patients were on a quite good metabolic control. In addition, we used RAPA and 30”STS tests because they are easy to be applied in the real-life setting. However, we appreciate that they have less sensibility and specificity than other methods exploring physical activity and capacity, and this may limit also the sensitivity of statistical analysis in a quite small group of patients.

Finally, we confirm that vitamin D deficiency is quite prevalent in PD patients. Treatment with Vitamin D may be useful in PD patients, where an increase of the functional capacity has been also reported [39].

## Conclusions

A high prevalence of low- performance capacity and sedentarism has been detected among elderly patients on PD or with CKD stage 3–4. Apart from age, a condition of malnutrition-inflammation was the major determinant of poor physical activity and capacity in PD patients. Better body composition seems to be associated with physical activity in PD and with physical capacity in CKD patients. Routine clinical management should include a close evaluation of nutritional status and evaluation of physical activity and capacity which can be easily assessed by RAPA and 30”STS tests.

## Abbreviations

APD: Automated peritoneal dialysis
BIA: Bio-impedance analysis
CAPD: Continuous peritoneal dialysis
CCI: Charlson comorbidity index
CKD: Chronic kidney disease
CNQA: Council of nutrition appetite questionnaire
eGFR: estimated glomerular filtration rate
ESRD: End-stage renal disease
GNRI: Geriatric nutritional risk index
IQR: Median and inter-quartile range (IQR)
MIS: Malnutrition-inflammation score
PD: Peritoneal dialysis
RAPA: Rapid assessment of physical activity
SD: Standard deviation
STS: Sit-to-stand
